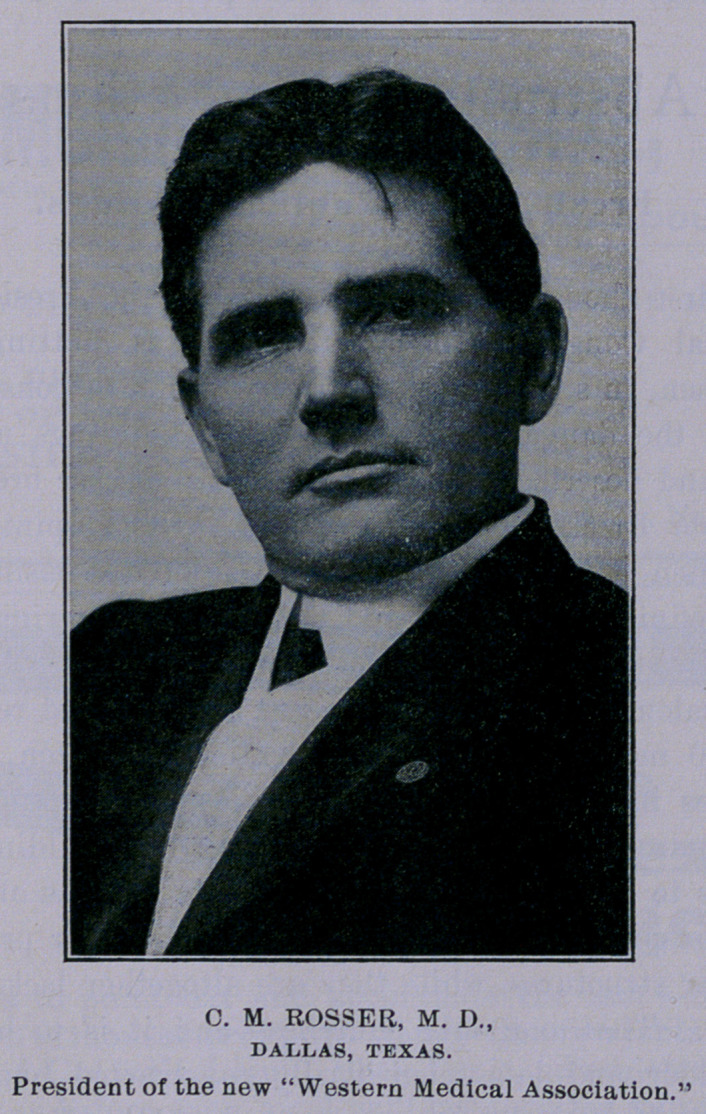# New Association of Southwest—Form of Organization Adopted by Physicians and Surgeons

**Published:** 1906-12

**Authors:** 


### Society Notes.

### New Association of Southwest—Form of Organization Adopted by Physicians and Surgeons.

The new medical association of the Southwest recently adjourned at Oklahoma City, it is predicted, will rank second only to the National body, the American Medical Association, and Dr. C. M. Rosser, its first President, is receiving the congratulations of his friends.

One feature of the organization was the merger of the Southwestern Tri-State Medical Society (Texas, Oklahoma and Indian Territory), an institution having been founded by leading physicians of Texas and the two Territories in this city six years ago. Several Dallas doctors had been honored by its offices, which were filled as follows: Presidents, Drs. H. K. Leake, John S. Turner, J. C. Loggins, W. R. Blailock, R. J. Crabell and George W. West, all except the last two from Texas; Secretaries, Drs. S. E. Milliken, Jno. O. McReynolds, Chas. M. Rosser and R. J. Crabell.

All of the annual meetings were held in Dallas except one, and the society was looked upon largely as a Dallas proposition to which the profession of the State was friendly.

When the two Territories were joined there was no longer an opportunity for a tri-State society as organized, and the new movement put into effect by a committee of five appointed by the President of the Societies of the Southwestern States included the original in its wider territory. A merger of the two logically resulted.

The *Oklahoman,* published at Oklahoma City, after giving an interesting account of the proceedings, has the following to say:

"The Constitution adopted at the meeting provides that physicians residing in Arkansas, Kansas, Missouri, Texas, and Oklahoma and Indian Territories, who are of good standing in their respective State societies, are eligible to membership and are enrolled upon payment of dues. The Association will meet in the fall of each year at a date to be determined upon by the Executive Committee.

"A Nominating Committee is formed by the selection of five members from each State, which, in turn selects three members from each State to be members of the Executive Committee. The Executive Committee transacts all business not of scientific character and directs the publication of papers. The scientific work is divided into four sections which shall name their respective chairmen and secretaries as follows:

"Medicine and allied branches; surgery and its specialties, eye, ear, nose and throat, and the strictly scientific subjects of anatomy, pathology and physiology.

"The conception of the plan rested with Jabez Jackson, of Kansas City, and was initiated by a committee appointed by the Presi-

C. M. ROSSER, M. D.,

DALLAS, TEXAS.

**President of the new "Western Medical Association."**

dent of the State Societies of the Southwest. The organization is not intended to interfere in any way with existing societies but on the other hand its promoters claim its purposes will be helpful to the parent body, the American Medical Association.

"C. M. Rosser, of Dallas, who was honored by an election to the office of President of the Association, is a native of Georgia, although he has resided in Texas since boyhood, and has been actively engaged in surgical practice in Dallas for a number of years. He has been first Vice-President of the Texas Medical Association and is at present Treasurer of the Southern Surgical and Gynecological Association. He is Professor of Surgery in the Baylor University

College of Medicine and Hospital Surgeon of the Baptist and other hospitals of Dallas. As founder of the Tri-State Medical Association of the Southwest he was instrumental in securing a union between the two societies, and his unanimous election yesterday attested the appreciation in which he is held by the profession representing the five States in the scope of the new and promising organization."

Hot Springs was selected as the next place of meeting.

## Figures and Tables

**Figure f1:**